# Comparative efficacy of different types of structured exercise interventions for idiopathic pulmonary fibrosis: a systematic review and meta-analysis

**DOI:** 10.3389/fmed.2026.1875852

**Published:** 2026-06-12

**Authors:** Wuzhen Wang, Yingying Gu, Lisha Mo

**Affiliations:** 1 School of Clinical Medicine, Jiangxi University of Chinese Medicine, Nanchang, Jiangxi, China; 2 School of Nursing, Jiangxi Medical College, Nanchang University, Nanchang, Jiangxi, China; 3 The Affiliated Hospital of Jiangxi University of Chinese Medicine, Nanchang, Jiangxi, China

**Keywords:** exercise, idiopathic pulmonary fibrosis, meta-analysis, mind-body integration training, structured multi-component training, systematic review

## Abstract

**Background:**

Exercise interventions have demonstrated favorable effects for patients with idiopathic pulmonary fibrosis, but the optimal exercise regimen remains unclear. To address this, we conducted a meta-analysis.

**Objective:**

Investigate the effects of different types of structured exercise interventions on patients with idiopathic pulmonary fibrosis and evaluate the quality of evidence for study outcomes using the GRADE system.

**Method:**

Search databases including PubMed, Embase, Web of Science, The Cochrane Library, and Scopus to collect randomized controlled trials (RCTs) on exercise interventions for idiopathic pulmonary fibrosis published from the inception of each database through December 2025. Analyses were performed using RevMan5.4.1and R4.4.2 software.

**Results:**

A total of 11 studies involving 503 patients were included. Meta-analysis results showed that exercise interventions significantly improved the 6-min walk distance (MD = 38.62 m, 95% CI: [25.72, 51.51], *P* < 0.00001) in exercise endurance, with mind-body integrated training showing potentially greater benefit. Regarding pulmonary function, mind-body integrated training significantly improved forced vital capacity (SMD = 0.76, 95% CI: [0.32, 1.20], *P* = 0.0008) and carbon monoxide diffusion capacity (SMD = 0.76, 95% CI: [0.42, 1.10], *P* < 0.0001). In contrast, structured multi-component training did not demonstrate consistent benefits across all pulmonary function measures. Regarding quality of life, mind-body integrated training demonstrated significant improvements across all dimensions and the total score of the St. George’s Respiratory Questionnaire, showing comprehensive efficacy; structured training proved effective only in certain dimensions. For dyspnea symptoms, mind-body integrated training exhibited significant improvement (SMD = −0.63, 95% CI: [−1.00, −0.27], *P* = 0.0006), whereas the effect of structured training was unclear and highly heterogeneous. Neither training approach demonstrated significant benefits for health status or psychological emotion wellbeing. Safety analysis was limited by incomplete adverse event reporting across studies; only one study provided detailed safety data, which showed no significant increase in adverse event risk with supervised exercise. GRADE evidence quality assessment revealed moderate-quality evidence for measures such as 6-min walk distance and carbon monoxide diffusion capacity, while most pulmonary function and quality of life indicators had low-quality evidence.

**Conclusion:**

Structured exercise interventions, including mind-body integrated training, can improve exercise endurance, selected pulmonary function measures, quality of life, and dyspnea symptoms in patients with idiopathic pulmonary fibrosis, but No firm conclusion on overall safety could be drawn. However, the evidence strength for most outcomes remains limited due to the risk of bias and heterogeneity in existing studies. Future high-quality research is needed to further validate the long-term benefits of different exercise modalities.

**Systematic review registration:**

https://www.crd.york.ac.uk/PROSPERO/view/CRD420251253143, CRD420251253143.

## Introduction

Idiopathic pulmonary fibrosis belongs to the spectrum of chronic, progressive interstitial lung diseases. Its essential feature is the replacement of normal lung parenchyma with dense scar tissue, leading to the gradual loss of the alveolar-capillary functional unit. Pathogenetically (1), persistent and recurrent alveolar epithelial injury drives abnormal repair processes. Multiple factors interact to amplify fibrotic signals, including activation of the inflammatory microenvironment, epithelial-mesenchymal transition (EMT), excessive extracellular matrix (ECM) deposition, endoplasmic reticulum stress, genetic predisposition, and environmental exposures. This ultimately leads to irreversible disruption of the gas exchange barrier. Among ILD subtypes (2), idiopathic pulmonary fibrosis (3) is the most representative. According to a study by Golchin et al. (4), the combined annual incidence rate of IPF is 5.8 per 100,000 (95% CI 4.8–6.8), with rates of 9.0 in North America, 5.1 in Europe, and 4.4 in Asia. The combined prevalence is 17.7 per 100,000, with rates of 27.2 in North America, 14.6 in Europe, and 14.8 in Asia. Moreover, the median survival for IPF is only 3–5 years (5), with a mortality rate surpassing most solid tumors. As population aging intensifies, the patient base continues to expand, gradually exceeding the threshold for classification as a “rare disease.”

Currently, treatment for idiopathic pulmonary fibrosis remains primarily focused on lung transplantation and supportive medications. In 2014, the US FDA successively approved two antifibrotic drugs: pirfenidone and nintedanib (6). Clinical evidence indicates (7) that both agents significantly reduce the annual decline rate of FVC, delay the progression of fibrosis, decrease the risk of worsening 6-min walk distance, postpone the first acute exacerbation, and tangibly improve patient disease trajectories. However, these drugs only act as “brakes”—they cannot reverse established fibrosis. High doses may induce gastrointestinal adverse reactions, and their efficacy is limited and single-dimensional (8). Moreover, the high cost of treatment makes it difficult for patients to afford. Studies by Diamantopoulos et al. (9), Zheng et al. (10), and others indicate that in North America, annual medical costs exceed $20,000 per year, while direct medical costs in China average $1,300.

Against the backdrop of high healthcare costs and the public’s demand for accessible, affordable solutions, exercise has increasingly demonstrated its advantages in preventing and treating major complex diseases. In recent years, various exercise training models have been progressively applied to the clinical intervention of pulmonary fibrosis, with related reports emerging steadily. Findings from Aktan et al. (11) indicate that an 8-week home-based remote rehabilitation program supplemented with IMT intervention improved inspiratory muscle strength, thereby enhancing functional exercise capacity and alleviating dyspnea. However, existing evidence primarily stems from small-sample, single-center randomized controlled trials, which are scattered and exhibit varying methodological quality. Therefore, this study aims to systematically review the latest randomized controlled trial evidence to clarify the efficacy and risks of different exercise modalities for patients with idiopathic pulmonary fibrosis, thereby recommending the most effective exercise intervention strategies for clinical practice.

## Materials and methods

This review was conducted in accordance with the PRISMA statement (Preferred Reporting Items for Systematic Reviews and Meta-Analyses) (12). The relevant research protocol has been submitted to the PROSPERO international systematic review prospective registration platform and assigned registration number CRD420251253143.

### Literature research

This study was conducted by two researchers (Wang and Gu) who independently performed literature searches. Data sources included multiple electronic databases such as PubMed, Embase, Web of Science, The Cochrane Library, and Scopus. The search timeframe spanned from the inception year of each database to December 2025. The literature search employed a combined strategy of medical subject headings (MeSH) and free-text terms, supplemented by manual tracing of reference lists from included studies to maximize the retrieval of relevant materials. Core keywords used in the search included: “Idiopathic Pulmonary Fibrosis,” “Interstitial Lung Disease,” “Pulmonary Fibrosis,” “Interstitial,” “exercise,” “physical activity,” and “fitness” among other corresponding English terms. Using PubMed search as an illustrative example, given the substantial volume of content, detailed search strategies for individual databases are presented in Supplementary Table 1.

### Inclusion and exclusion criteria

(1) Patients with confirmed idiopathic pulmonary fibrosis (13) meeting international diagnostic criteria, regardless of gender, age, ethnicity, or disease duration; (2) The intervention group receives structured exercise therapy (including but not limited to aerobic, resistance, high-intensity interval, respiratory muscle training, or traditional Chinese exercises), which may be combined with conventional drug therapy; (3) The control group receives all treatments identical to the intervention group except for exercise therapy; (4) Outcome measures: 1. 6MWD; 2. Pulmonary function (DLCO, FEV1, FVC); 3. SGRQ scores (Activity, Impact, Symptoms, Total); 4. K-BILD, Psychological Emotion; 5. Dyspnea Symptoms; 6. Incidence rate of adverse reactions; (5) Published randomized controlled trials (RCT).

(1) Presence of exercise contraindications (e.g., recent myocardial infarction, unstable angina, uncontrolled severe arrhythmias, etc.); (2) Unstructured intervention protocols: trial groups receiving only verbal advice such as “increase daily activity” or lacking systematic exercise prescriptions with defined intensity, frequency, duration, and progression principles; (3) Inconsistent outcome measurement tools or methods, precluding data pooling or comparison. (4) Inappropriate study design: Non-randomized controlled trials (e.g., observational studies, case series, reviews, meta-analyses), or studies not published in full text (e.g., conference abstracts, research protocols, trial registrations).

When duplicate or overlapping data were found across multiple reports, only the most comprehensive version was included.

### Data extraction

Two authors (Wang and Gu) independently screened the included literature and collected relevant data using a self-designed data extraction form. Disagreements during the assessment process were resolved through discussion and consensus reached with the involvement of a third reviewer. The extracted information primarily included: first author, publication year, country, study design type, implementation location, subject characteristics (sample size, age), intervention protocol (specific format, duration, implementation frequency), and various outcome measures. The mean and standard deviation of each measure were also recorded for subsequent effect size calculations.

### Risk of bias assessment

Two reviewers (Wang and Gu) independently assessed the methodological quality of the final included studies using the Cochrane Risk of Bias tool 2.0 (ROB2.0). This tool assessed each study across five dimensions: randomization process, intervention deviation, completeness of outcome data, outcome measurement methods, and reporting of results. An overall rating of “low risk,” “high risk,” or “risk of bias unclear” was assigned. In cases of disagreement between reviewers, a third researcher was consulted to facilitate discussion and reach a final consensus.

### Statistical analysis

All statistical analyses were performed using RevMan 5.4.1 and R 4.4.2 software. For continuous outcome measures, if studies employed different measurement tools, standardized mean differences (SMD) were calculated for pooling; if the same measurement tools were used, mean differences (MD) were directly used for effect size synthesis. Pooled analyses were based on either random-effects or fixed-effects models (14), with 95% confidence intervals reported.

Heterogeneity between studies was assessed using the I^2^ statistic (15). An I^2^ value > 50% was considered indicative of substantial heterogeneity. Specifically, I^2^ values between 25% and 50% indicate low heterogeneity, 50%–75% indicate moderate heterogeneity, and values exceeding 75% indicate high heterogeneity (16). If the number of included studies is sufficient, subgroup analyses will be conducted to explore potential sources of heterogeneity. The statistical significance level was set at *P* < 0.05.

To clarify the differences in treatment efficacy among various exercise modalities for patients with idiopathic pulmonary fibrosis, this study categorized the included literature into the following two subgroups based on the structural characteristics of the intervention measures:

(1) Structured multi-component training: Integrated exercise programs incorporating two or more training modalities (e.g., aerobic combined with resistance training);

(2) Mind-body integration training: In this systematic review, mind-body integrated training refers to exercise interventions that explicitly incorporate at least two of the following three core components: (1) breathing regulation (e.g., diaphragmatic breathing, pursed-lip breathing); (2) postural control with slow, coordinated movements (e.g., yoga asanas, Baduanjin, Dao yin); and (3) mental concentration or meditation guidance (e.g., body awareness, visualization, relaxation).

We acknowledge that specific practices such as yoga, Baduanjin, and breathing rehabilitation differ in their relative emphasis on movement versus breathing. However, they share the therapeutic principle of combining physical, psychological, and respiratory components. Therefore, grouping them together in the primary analysis aims to generate a practical hypothesis: whether training incorporating mind-body elements may confer additional value compared to structured multicomponent training.

## Results

### Search process

**FIGURE 1:**
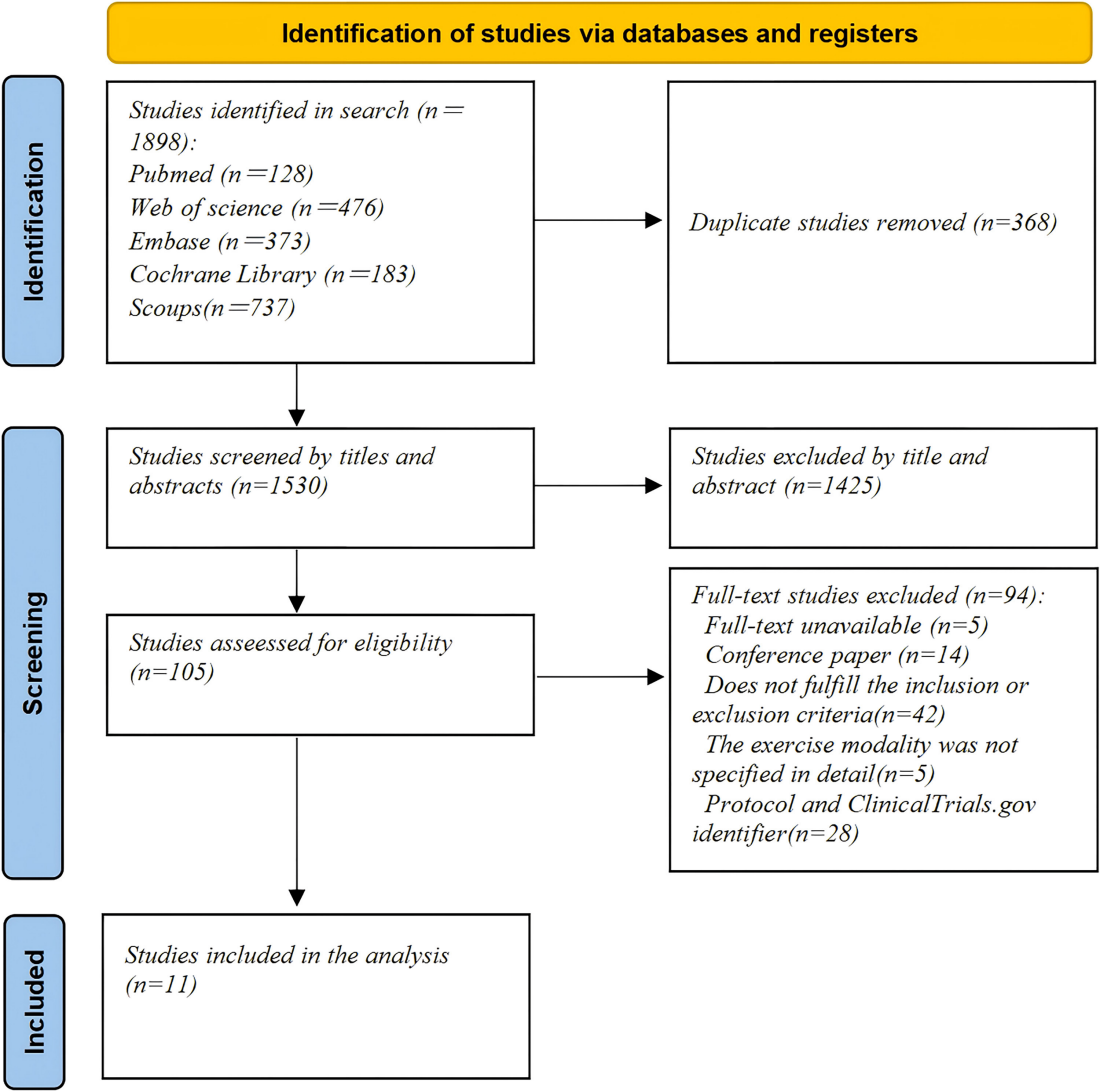
Screening process.

As shown in Figure 1, the initial search yielded 1,898 records distributed across PubMed (128), Web of Science (476), Embase (373), Cochrane Library (183), and Scopus (737). After removing 368 duplicates, 1,530 records underwent title/abstract screening, resulting in the exclusion of 1,425 articles. Full texts of 105 studies underwent eligibility assessment, with 94 ultimately excluded: 5 full texts were unavailable, 14 were conference abstracts, 42 did not meet inclusion/exclusion criteria, 5 had unclear exercise protocols, and 28 contained only protocols or ClinicalTrials.gov registration numbers. Ultimately, 11 studies were included in this systematic review and meta-analysis.

### Study characteristics

As shown in Table 1, the 11 included studies were published between 2007 and 2025, covering multiple countries including the United States (3 studies), China (2 studies), Japan (2 studies), Turkey (1 studies), Denmark (1 studies), Germany (1 studies), and Israel (1 studies). The studies by Jackson et al. (17) and Gaunaurd et al. (18) originated from the same research team. All trials were conducted at medical centers, university-affiliated hospitals, or specialized outpatient clinics, enrolling a total of 503 patients with idiopathic pulmonary fibrosis. Sample sizes ranged from 21 to 88 patients, with most participants aged between 60 and 75 years.

**TABLE 1:** Characteristics of included studies.

References	Design	Setting	Number of patients (EG/CG)	Age (year)	Experimental group			Control group	Outcome
					Invention type	Frequency/duration and intensity	Duration		
Kadura et al. (20) USA	RCT	The Center for Interstitial Lung Diseases at the University of Washington Medical Center	60IPF 30EG 30CG	EG: 72.5 (8.97) CG: 74.3 (7.41)	Yoga program	Sessions lasted one hour, twice/week	12 consecutive weeks	Standard of care and following usual activities	L-IPF, K-BILD, HADS
Atmaca et al. (21) Turkey	RCT	Istanbul University Cerrahpaşa Medical Faculty, Department of Chest Diseases	28IPF 14EG 14CG	EG: 61.64 (8.01) CG: 59.00 (7.32)	Baduanjin	Sessions lasted one hour, thrice/week	8 consecutive weeks	Following usual activities	6MWD (m), FVC (% predicted), FEV1 (% predicted), SGRQ-Symptom, SGRQ-Activity, SGRQ-Impact, SGRQ-Total
Kataoka et al. (22) Japan	RCT	Medical institution	88IPF 45EG 43CG	EG: 71.2 (4.9) CG: 70.4 (5.5)	Endurance training, walking, resistance training	Sessions lasted half hour, twice/week	12 consecutive weeks	Standard of care	6MWD (m), FVC
Zhou et al. (23) China	RCT	The First Affiliated Hospital of Henan University of Chinese Medicine	63IPF 32EG 31CG	EG: 66 (11) CG: 67 (10)	Pulmonary daoyin	Sessions lasted one hour, five times/week	8 consecutive weeks	Following usual activities	6MWD (m), mMRC, FVC (L), DLCO (% predicted), SGRQ-Symptoms, SGRQ-Activity, SGRQ-Impact, SGRQ-Total
Shen et al. (19) China	RCT	The Department of Respiratory Medicine, Shanghai Pulmonary Hospital, Tongji University	82IPF 39EG 43CG	EG: 65.31 (6.11) CG: 64.95 (7.97)	LHP’s respiratory rehabilitation	Sessions lasted A quarter of an hour, seven times/week	48 consecutive weeks	Standard of care and following usual activities	SGRQ-Total, 6MWD (m), FVC (L), FEV1 (L), DLCO (%)
Cerdán-de-las-Heras et al. (24) Denmark	RCT	The outpatient clinic at Center for Rare Lung Diseases, Department of Respiratory Diseases and Allergy, Aarhus University Hospital, Denmark.	29IPF 15EG 14CG	EG: 70.1 (8.8) CG: 72.4 (7.6)	Elastics, weights and a fitness-step	Sessions lasted 10–20 min, three-five times/week	12 consecutive weeks	Usual care	6MWD (m), SGRQ-Total, GAD7, k-BILD
Jarosch et al. (25) Germany	RCT	Hospital of the Schön Klinik Berchtesgadener Land	51IPF 34EG 17CG	EG: 68 (9) CG: 65 (10)	Endurance or interval cycle training, resistance training	Five-six times/week	3 consecutive weeks	Usual care	6MWD (m)
Vainshelboim et al. (26) Israel	RCT	The Rabin Medical Center, Beilinson Hospital, Petah Tikva, Israel.	32IPF 15EG 17CG	EG: 68.8 (6) CG: 66 (9)	Aerobic, resistance, and flexibility exercise, breathing exercises	Sessions lasted half hour, twice/week	12 consecutive weeks	Regular medical care	FVC % predicted, DLCO % predicted, 6MWD (m), mMRC (0–4), SGRQ-Total, SGRQ-Symptoms, SGRQ-Impact, SGRQ-Activity
Jackson et al. (17) USA	RCT	VA Medical Center, Miami	21IPF 11EG 10CG	EG: 71 (6) CG: 66 (7)	Treadmill walking, semirecumbent cycling, self-administered standing or seated with home exercise program	Sessions lasted two hours, twice/week	12 consecutive weeks	Usual care	6MWD (m), Dyspnea
Gaunaurd et al. (18) USA	RCT	VA Medical Center, Miami	21IPF 11EG 10CG	EG: 71 (6) CG: 66 (7)	Treadmill walking and recumbent cycling, flexibility exercises, strength training	Sessions lasted 1.5 h, twice/week	12 consecutive weeks	Following usual activities	SGRQ-Symptom
Nishiyama et al. (27) Japan	RCT	Outpatient clinic of Department of Respiratory Medicine and Allergy, Tosei General Hospital	28IPF 13EG 15CG	EG: 68.1 (8.9) CG: 64.5 (9.1)	Treadmill walking, Strength training using elastic bands, arm raising and knee extensions	Sessions lasted 50 min, twice/week	10 consecutive weeks	Usual care	FVC (L), FEV1 (L), 6MWD (m), BDI score, SGRQ-Symptoms, SGRQ-Activity, SGRQ-Impacts, SGRQ-Total

Interventions were primarily categorized into two types: structured comprehensive training (7 studies) centered on combinations of aerobic, resistance, endurance, and flexibility exercises; and mind-body integration training (4 studies) encompassing yoga, Baduanjin, traditional Chinese guided exercises, and specialized respiratory rehabilitation training. Exercise frequency typically ranged from 2 to 5 sessions per week, with individual session durations spanning 10 min to 2 h. Intervention cycles generally lasted 8 to 12 weeks, though Shen et al. (19) conducted a study extending up to 48 weeks. Control groups primarily received routine medical care or maintained daily activities without systematic exercise intervention.

Outcome measures focused on exercise capacity (6MWD, 9 items), lung function (FVC, FEV1, DLCO, 6 items), quality of life (SGRQ and its subscales, 7 items), dyspnea scores (L-IPF/mMRC/Dyspnea/BDI, 5 items), psychological emotion scales (HADS, GAD-7, 2 items), and disease-specific quality of life questionnaires (K-BILD, L-IPF, 2 items). Overall, the included studies demonstrated good clinical consistency in intervention classification and outcome measures, providing a foundation for subsequent meta-analysis.

### Risk of bias

**FIGURE 2:**
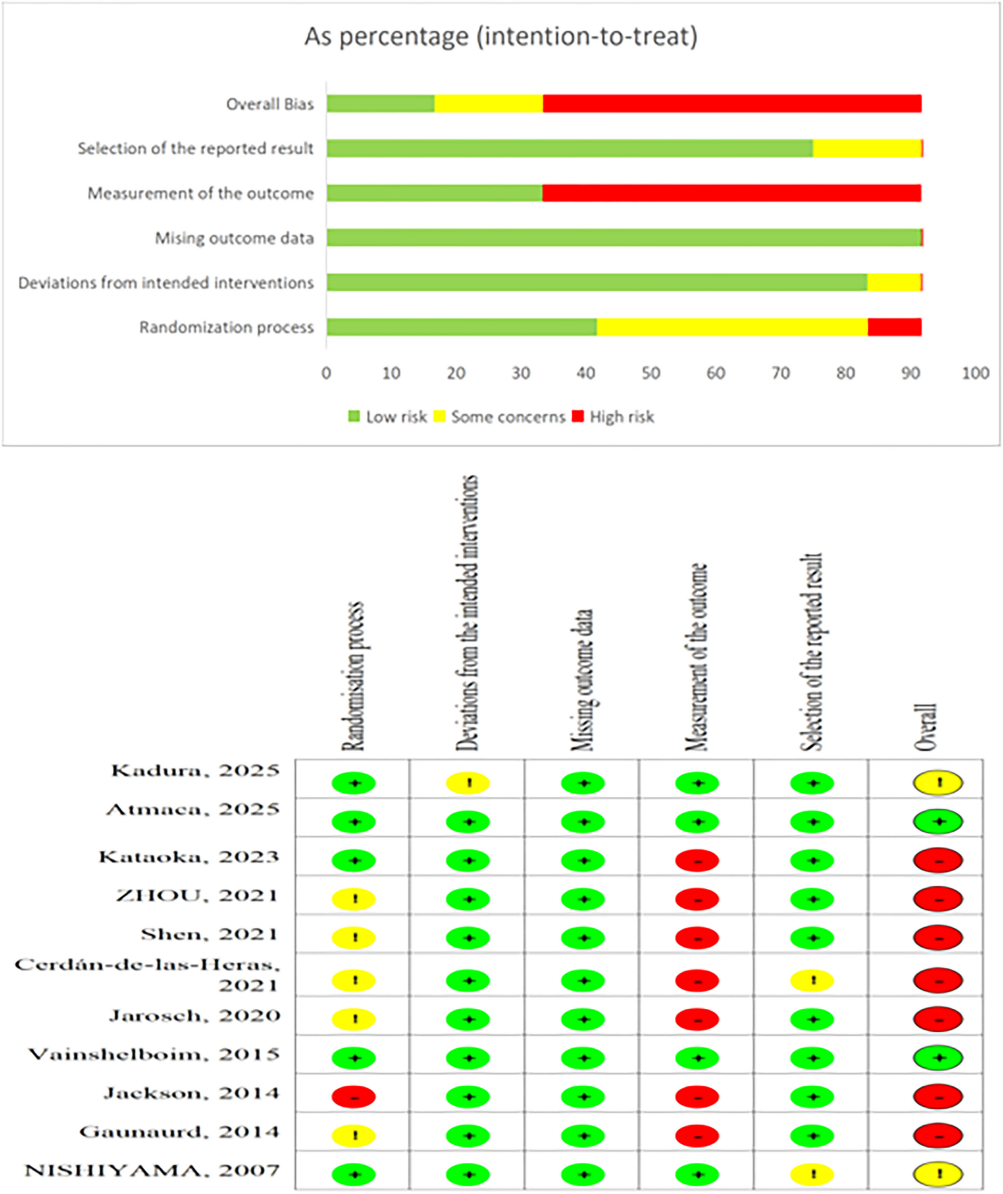
Summary table of risk of bias assessment.

As shown in Figure 2, the risk of bias in the included studies is summarized.

Regarding selection bias (randomization process), all included studies reported random assignment. Five studies (20–22, 26, 27) explicitly reported using computer-generated random sequences (e.g., block randomization, online randomization systems) combined with sealed envelopes, electronic data capture (EDC) systems, or independent personnel managing the allocation sequence, indicating a low risk of bias. Four studies (18, 19, 24, 25) mentioned randomization (e.g., random number tables, block randomization) but did not adequately describe allocation concealment mechanisms, raising some concern about bias risk. Among the two studies (17, 23), one of them (17) was rated as high risk due to explicitly not implementing allocation concealment, while Another one (23) was rated as “some concern” due to unclear reporting of random sequence generation methods.

Regarding intervention deviation, one study (20) explicitly stated that the intervention was unblinded and was rated as “some concern,” while the others were rated as low risk.

Regarding attrition bias (missing data), 10 studies (17–19, 21–27) demonstrated low risk of bias due to intention-to-treat (ITT) analysis or low loss-to-follow-up rates (≤ 16%). One study (20) had minimal loss-to-follow-up despite some attrition and was still rated “low risk.” No studies were identified as high risk due to excessively high loss-to-follow-up rates (e.g., > 40%).

Regarding detection bias (outcome measurement), since non-blinding may influence subjective evaluation results, four studies (20, 21, 26, 27) explicitly employed outcome assessor blinding, indicating a low risk of bias. Seven studies (17, 22, 25) explicitly reported that assessors were not blinded, while (18, 19, 23, 24) did not mention blinding information and were judged to have a high risk of bias.

Regarding selective reporting bias, findings varied significantly: 9 studies (17–23, 25, 26) were prospectively registered on clinical trial platforms and reported prespecified outcomes with high completeness, indicating low risk of bias; Two studies (24, 27) were rated as having “some concern” for bias risk due to lack of registration or inadequate reporting of all pre-specified outcomes.

In summary, the assessor’s overall judgment indicates that 2 studies (21, 26) are at low risk, 2 studies (20, 27) raise some concerns, and 7 studies (17–19, 22–25) are at high risk, suggesting considerable heterogeneity in the methodological quality of the included evidence.

### Effects of different types of structured exercise interventions on patients with idiopathic pulmonary fibrosis

#### 6 min walk distance (6 MWD)

**FIGURE 3:**
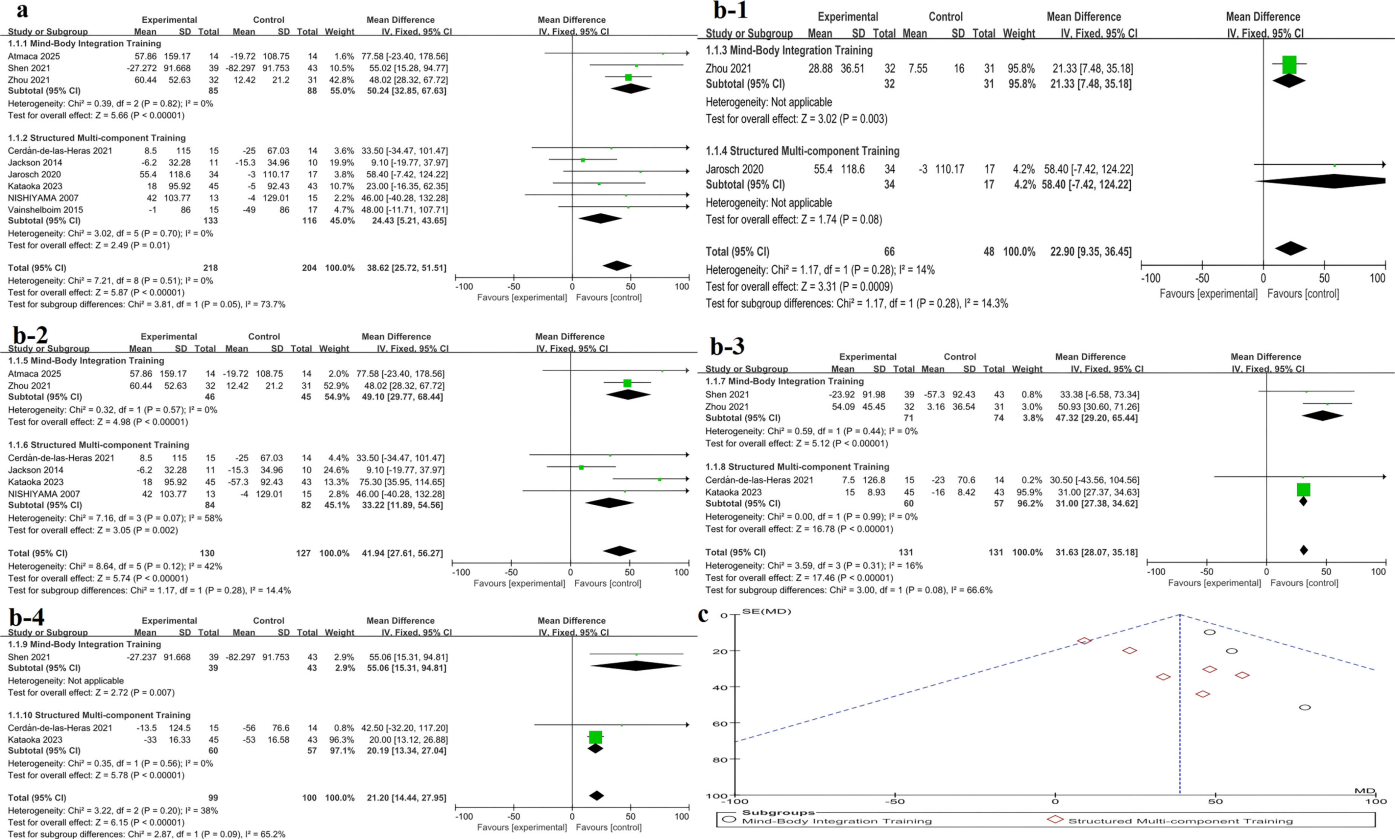
**(a)** Forest plot: Effects of exercise training on 6-min walk distance. **(b-1)** Forest plot: Effects of exercise training on 6-min walk distance (≤ 4 weeks). **(b-2)** Forest plot: Effects of exercise training on 6-min walk distance (> 4 weeks ≤ 12 weeks). **(b-3)** Forest plot: Effects of exercise training on 6-min walk distance (> 12 weeks ≤ 26 weeks). **(b-4)** Forest plot: Effect of exercise training on 6-min walk test change (> 26 weeks). **(c)** Funnel plot: Effect of exercise training on 6-min walk distance.

As shown in Figure 3, Data were extracted according to the exercise training schedule. A total of 9 studies (involving 422 participants) examined the effect of exercise training on the 6-min walk distance. The meta-analysis results, as shown in the figure, indicate that exercise programs significantly improve patients’ exercise endurance. The analysis demonstrated a low overall risk of publication bias (funnel plot showing broadly symmetrical data points) and low heterogeneity among included studies (total I^2^ = 0%), enhancing the reliability of the findings. The pooled analysis indicated that rehabilitation programs significantly increased the mean 6MWD by 38.62 meters (95% CI: [25.72, 51.51]; *P* < 0.00001). Subgroup analyses suggested that the effectiveness may differ across training modalities. Mind-body integrative training was associated with greater improvement in 6-min walk distance (MD = 50.24 m [95% CI 32.85, 67.63]), while structured multicomponent training also showed improvement (MD = 24.43 m [95% CI 5.21, 43.65]). However, the test for subgroup differences only reached borderline statistical significance (*P* = 0.05, I^2^ = 73.7%). Given that these between-subgroup comparisons constitute indirect comparisons, the above results should be regarded as hypothesis generating findings rather than conclusive evidence. Future trials with direct comparisons are needed to verify whether certain training modalities are indeed superior to others. In summary, these findings suggest that rehabilitation programs may improve patients’ walking ability, although the high risk of bias in most included studies limits the certainty of this conclusion.

Additionally, we conducted a meta-analysis based on different intervention durations. Results indicated that rehabilitation programs demonstrated significant positive effects at all time points, though the magnitude and pattern of effects varied over time.

A total of 2 studies involving 144 participants (≤ 4 weeks) showed that rehabilitation programs significantly improved 6MWD (MD = 22.90 meters, 95% CI: [9.35, 36.45], *P* = 0.0009). However, studies in this timeframe were limited, and subgroup differences were not significant (*P* = 0.28).

A total of 6 studies involving 257 participants (4–12 weeks) showed more pronounced effects, with an overall effect size of MD = 41.94 m, 95% CI: [27.61, 56.27], (*P* < 0.00001). Among these, mind-body integration training showed an effect size of MD = 49.10, 95% CI: [29.77, 68.44], which remained higher than structured multi-component training (MD = 33.22, 95% CI: [11.89, 54.56]). However, the difference between subgroups did not reach statistical significance (*P* = 0.28).

A total of 4 studies involving 262 participants (12–16 weeks): Rehabilitation programs remained significantly effective with MD = 31.63 m, 95% CI: [28.07, 35.18] (*P* < 0.00001). The effect of mind-body integration training at this stage (MD = 47.32 m, 95% CI: [29.20, 65.44]) remained higher than that of structured multi-component training (MD = 31.63 m, 95% CI: [27.38, 34.62]), but the difference between subgroups remained non-significant (*P* = 0.08).

Three studies involving 199 participants (> 26 weeks): Effects were sustained and significant (MD = 21.20 m, *P* < 0.00001). The long-term effect point estimate for mind-body integrated training was higher (MD = 55.06 m), but the subgroup difference compared to structured multicomponent training (MD = 20.19 m) remained borderline (*P* = 0.09, I^2^ = 65.2%).

Exercise training demonstrates sustained and significant efficacy in improving 6MWD, with effects potentially peaking at the mid-term (4–12 weeks). Subgroup analysis suggests that mind-body integrated training exhibits larger effect estimates at most time points, particularly during long-term follow-up; however, differences between training modalities did not reach conventional statistical significance at any time point (all *P*-values > 0.05), indicating both are effective rehabilitation strategies. Study heterogeneity was generally low across all analysis phases (I^2^ < 50% in most cases), supporting the consistency of results.

### Pulmonary function

**FIGURE 4:**
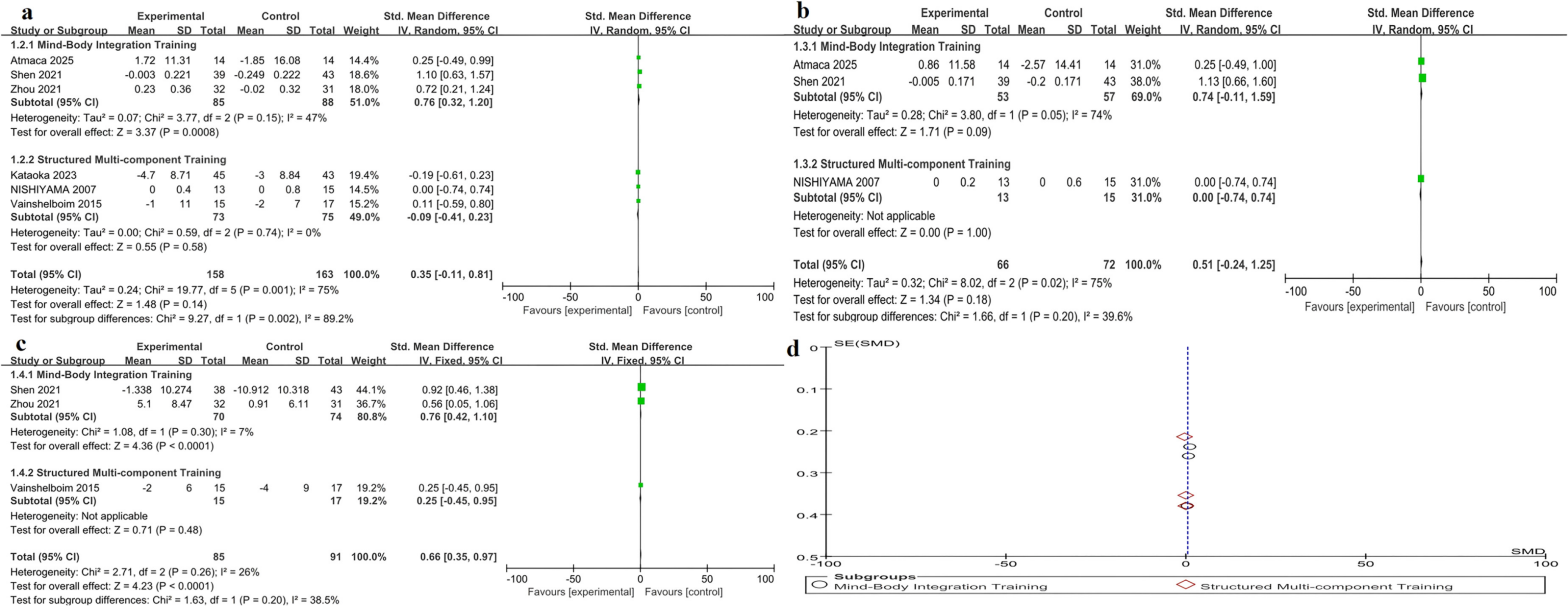
**(a)** Forest plot: Effects of exercise training on forced vital capacity. **(b)** Forest plot: Effects of exercise training on carbon monoxide diffusion capacity. **(c)** Forest plot: Effects of exercise training on forced expiratory volume in 1 s. **(d)** Funnel plot: Effects of exercise training on forced vital capacity.

As shown in Figure 4, a meta-analysis of forest plots for three respiratory function indicators (FVC, FEV1, DLCO) systematically compared the effects of mind-body integration training versus structured multi-component training on respiratory function. Six studies involving 321 participants examined FVC, three studies involving 138 participants examined FEV1, and three studies involving 176 participants examined DLCO. Results demonstrated that mind-body integrated training significantly improved both forced vital capacity and carbon monoxide diffusion capacity (FVC subgroup SMD = 0.76, 95% CI: [0.32, 1.20], *P* = 0.0008; DLCO subgroup SMD = 0.76, 95% CI: [0.42, 1.10], *P* < 0.0001), whereas structured multicomponent training failed to demonstrate consistent significant benefits across all measures. However, neither training approach yielded significant improvements in forced expiratory volume in 1 s (FEV1) (overall *P* = 0.18). Notably, high heterogeneity existed across studies (particularly for FVC and FEV1, with I^2^ reaching 75%). Funnel plots indicated potential publication bias in existing evidence regarding exercise training’s impact on FVC, which may overestimate the overall intervention effect.

Due to the limited sample size and the absence of long-term follow-up in most studies involving pulmonary function measurements, it is challenging to conduct a meta-analysis using training duration as a cutoff point. Moreover, given that most of these pulmonary function outcomes were derived from studies with high risk of bias (as shown in Figure 2), the observed improvements should be interpreted with caution.

### St George’s respiratory questionnaire

**FIGURE 5:**
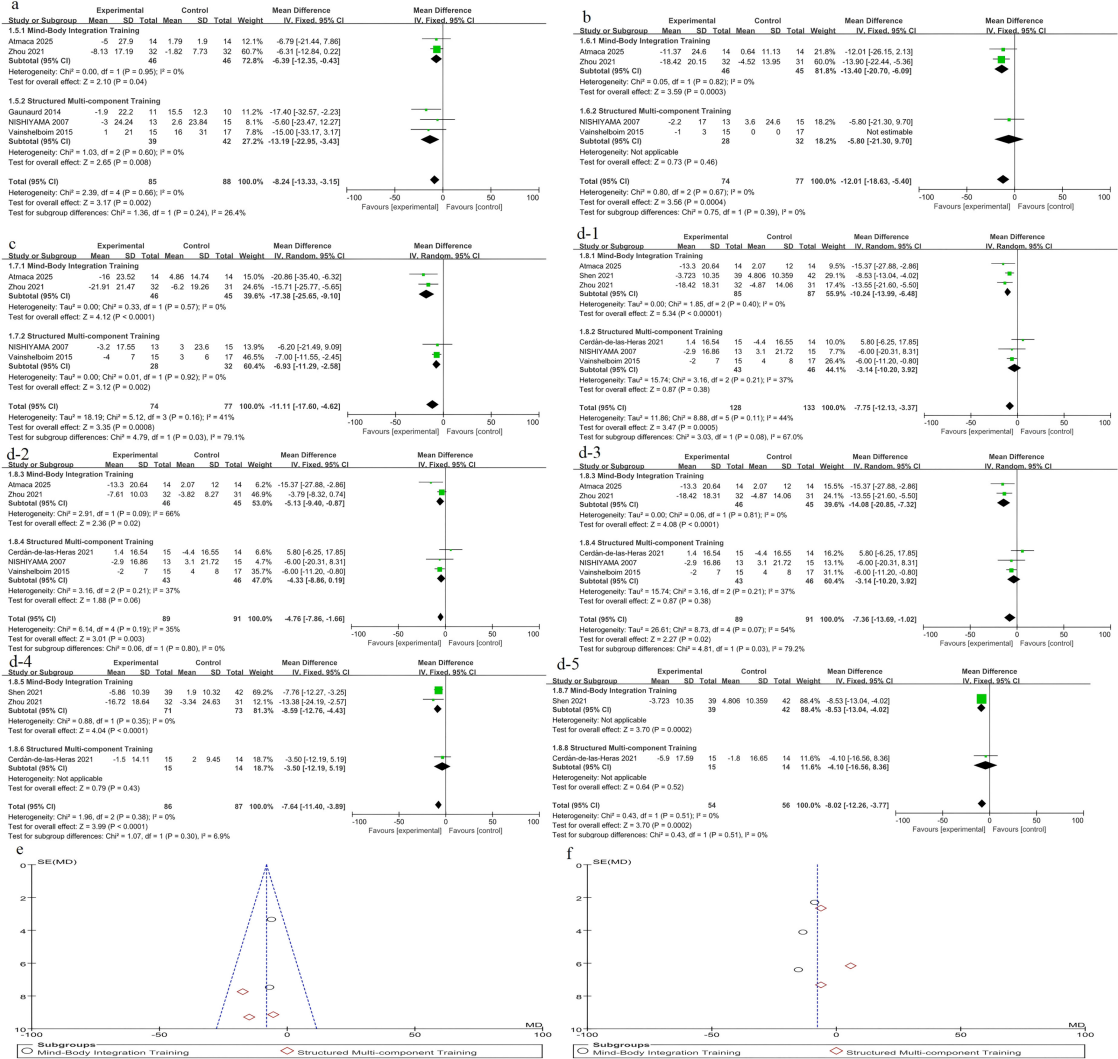
**(a)** Forest plot: Effects of exercise training on SGRQ-Symptom. **(b)** Forest plot: Effects of exercise training on SGRQ-Activity. **(c)** Forest plot: Effects of exercise training on SGRQ-Impact. **(d-1)** Forest plot: Effects of exercise training on SGRQ-Total. **(d-2)** Forest plot: Effects of exercise training on SGRQ-Total (< 12 weeks; Shen et al. (19) SGRQ-Total data reported at 4 weeks). **(d-3)** Forest plot: Effect of exercise training on SGRQ-Total (< 12 weeks; Shen et al. (19) SGRQ-Total data at 8 weeks). **(d-4)** Forest plot: Effect of exercise training on SGRQ-Total (> 12 weeks ≤ 24 weeks). **(d-5)** Forest plot: Effect of exercise training on SGRQ-Total (> 24 weeks). **(e)** Funnel plot: Effects of exercise training on SGRQ-Symptom. **(f)** Funnel plot: Effects of exercise training on SGRQ-Total.

As shown in Figure 5, a meta-analysis of forest plots for the four dimensions of the St. George’s Respiratory Questionnaire (Activity, Impact, Symptom, Total) we systematically compared the effects of mind-body integration training versus structured multicomponent training on quality of life in patients with respiratory diseases. Regarding SGRQ-Activity, four studies involving 151 participants were included; for SGRQ-Impact, four studies involving 151 participants were included; for SGRQ-Symptom, five studies involving 173 participants were included; and for SGRQ-Total, six studies involving 261 participants were included. Results indicate that mind-body integration training significantly improved SGRQ-Activity (MD = −13.40, 95% CI: [−20.70, −6.09], *P* = 0.0003), SGRQ-Impact (MD = −17.38, 95% CI: [−25.65, −9.10], *P* < 0.0001), SGRQ-Symptom (MD = −6.39, 95% CI: [−12.35, −0.43], *P* = 0.04), and SGRQ-Total (MD = −10.24, 95% CI: [−13.99, −6.48], *P* < 0.00001). while structured multi-component training showed significant effects only on SGRQ-Impact (MD = −6.93, 95% CI: [−11.29, −2.58], *P* = 0.002) and SGRQ-Symptom (MD = −13.19, 95% CI: [−22.95, −3.43], *P* = 0.008), showing no significant benefits in activity capacity or total scores. It is noteworthy that the heterogeneity among studies was generally low (most I^2^ = 0%), suggesting consistent results within similar training categories. However, funnel plots indicate potential publication bias in SGRQ-Symptom and SGRQ-Total scores, which may lead to an overestimation of the overall effect. Overall, mind-body integration training showed statistically significant improvements in all SGRQ domains, whereas the effects of structured multi-component training were limited to specific domains. However, given the indirect nature of this cross-study comparison and the lack of significant subgroup differences in most time-point analyses, these findings should be considered exploratory.

Given the limited sample size across SGRQ dimensions, our further meta-analysis of SGRQ-Total scores across different intervention durations demonstrated that pulmonary rehabilitation programs consistently yielded significant benefits in improving respiratory-related quality of life, with the pattern of effects exhibiting certain temporal variations.

Short-term interventions (≤ 12 weeks): Five studies involving 180 participants were included. Rehabilitation programs significantly reduced SGRQ total scores in the short term (pooled MD = −4.76, 95% CI: [−7.86, −1.66], *P* = 0.003), indicating rapid improvement in quality of life. However, no significant differences were observed between subgroups during this period (*P* = 0.80).

Mid-term intervention (12–24 weeks): Three studies involving 173 participants were included. Rehabilitation benefits remained significant and more stable (pooled MD = −7.64, 95% CI: [−11.40, −3.89], *P* < 0.00001). Point estimates differed between mind-body integrated training (MD = −8.59) and structured multi-component training (MD = −3.50), but subgroup comparisons remained non-significant (*P* = 0.30).

Long-term intervention (> 24 weeks): Two studies involving 110 participants were included. The positive effects of rehabilitation were sustained (pooled MD = −8.02 points, 95% CI: [−12.26, −3.77], *P* = 0.0002). At this stage, mind-body integrated training showed a larger point estimate (MD = −8.53 points), but subgroup differences compared with structured multicomponent training (MD = −4.10 points) remained non-significant (*P* = 0.51).

In summary, exercise training demonstrated sustained and significant efficacy in improving SGRQ scores. Although point estimates for mind-body integrated training tended to be larger at most time points, differences between it and structured multicomponent training failed to reach conventional statistical significance (*P* > 0.05) across all time periods, suggesting both rehabilitation models are effective strategies. Except for moderate heterogeneity in the short-term analysis (I^2^ = 54%), the medium- and long-term analyses demonstrated low between-study heterogeneity (I^2^ = 0%), supporting the consistency of results across different studies.

### Multi-scale (L-IPF/mMRC/Dyspnea/BDI) comprehensive assessment of dyspnea

**FIGURE 6:**
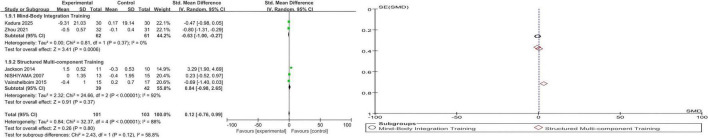
Forest plot: Effects of exercise training on dyspnea symptoms. Funnel plot: Effects of exercise training on dyspnea symptoms.

As shown in Figure 6, a meta-analysis combining dyspnea scores from four scales—L-IPF, mMRC, Dyspnea, and BDI—using standardized mean differences (SMD) systematically compared the effects of mind-body integrated training versus structured multicomponent training on improving dyspnea symptoms in patients. The analysis included five studies involving 204 participants.

Results demonstrated that mind-body integrated training showed significant efficacy in alleviating dyspnea (subgroup SMD = −0.63, 95% CI: [−1.00, −0.27], *P* = 0.0006), with no heterogeneity among studies (I^2^ = 0%). Structured multi-component training showed no significant benefit (subgroup SMD = 0.84, 95% CI: [−0.98, 2.65], *P* = 0.37), with extremely high heterogeneity among studies within this subgroup (I^2^ = 92%), indicating inconsistent results. The overall pooled effect did not reach statistical significance (SMD = 0.12, 95% CI: [−0.76, 0.99], *P* = 0.80), with high overall heterogeneity (I^2^ = 88%). The funnel plot exhibited asymmetry, suggesting potential publication bias in the evidence.

Overall, mind-body integrated training was associated with a consistent positive effect on alleviating dyspnea symptoms. At the same time, the effects of structured multi-component training were less consistent, with variation across studies. It should be noted that these findings are derived from indirect subgroup comparisons and more likely reflect differences in study populations, intervention protocols, or dyspnea measurement methods. Given the indirect nature of the evidence and the substantial heterogeneity across studies, the difference in effectiveness between the two training modalities requires further investigation.

### K-BILD, psychological emotion

**FIGURE 7:**
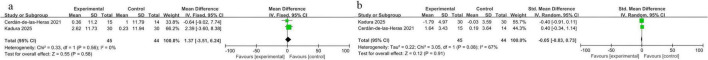
Forest plot **(a)**: Effects of exercise training on K-BILD. Forest plot **(b)**: Effects of exercise training on psychological emotion.

As shown in Figure 7, this section assessed the impact of interventions on patients’ health status and psychological symptoms through a combined analysis of the K-BILD total score and anxiety/depression scales (HADS and GAD-7). The two analyses encompassed four studies (two evaluating K-BILD, two evaluating anxiety/depression), involving a total of 89 participants. Regarding the K-BILD total score (using mean difference MD), the pooled analysis showed no significant improvement from the interventions (MD = 1.37, 95% CI: [−3.51, 6.24], *P* = 0.58), with no heterogeneity between studies (I^2^ =0%). Regarding psychological and emotional outcomes (anxiety and depression symptoms) measured using standardized mean differences (SMD), the meta-analysis similarly failed to demonstrate significant effects (SMD = −0.05, 95% CI: [−0.83, 0.73], *P* = 0.91). However, moderate heterogeneity existed between studies (I^2^ = 67%), suggesting potential inconsistencies in results across different research investigations. Overall, existing evidence indicates that current interventions show no significant benefit in improving patients’ K-BILD overall health status or anxiety and depression symptoms; notably, assessment results for anxiety and depression symptoms varied considerably across studies.

### Incidence rate of adverse reactions

Among the 11 included RCTs, only the study by Kataoka et al. (22) systematically reported adverse events. In that study, all patients received nintedanib as standard of care. The reported adverse events elevated liver enzymes (27% in the rehabilitation group vs. 21% in the control group, *P* = 0.62), anorexia (24% vs. 16%, *P* = 0.61), and nausea (20% vs. 9%, *P* = 0.38) are known side effects of nintedanib rather than complications of the exercise intervention itself. No significant between-group differences were observed for these events, suggesting that adding supervised exercise did not increase the frequency of drug-related adverse events. For severe events (acute exacerbation of IPF, mortality, IPF progression, bleeding) and cardiovascular events (deep vein thrombosis, acute coronary syndrome, stroke, arrhythmia), no significant between-group differences were found (all *P* > 0.05). However, due to the lack of adverse event reporting in the remaining 10 studies, the safety profile of other rehabilitation modalities could not be adequately assessed. Rehabilitation training is recommended under supervision and with supplemental oxygen as needed.

### Quality of evidence

As shown in Table 2, Indicators with “moderate” evidence quality include: 6MWD, DLCO, SGRQ-Activity, SGRQ-Symptom, K-BILD-Total. These findings are relatively robust, suggesting that interventions may have positive implications for improving patients’ exercise endurance, certain pulmonary function measures, and specific quality-of-life dimensions. They may serve as a reference for clinical practice and decision-making. Indicators with “low” evidence quality include: FVC, FEV1, SGRQ-Impact, SGRQ-Total, Dyspnea Symptoms, and Psychological Emotion. These findings are constrained by high risk of bias and heterogeneity, and the current evidence is insufficient to draw definitive conclusions. Interpretation requires extreme caution. Overall, this study suggests that mind-body integration training shows potential for certain outcomes. However, the overall strength of evidence is limited due to methodological quality and heterogeneity across included studies. Future research requires larger-scale, methodologically rigorous randomized controlled trials with blinding in both implementation and assessment to further validate the intervention’s effectiveness and elevate the level of evidence.

**TABLE 2:** Quality of evidence.

Outcome	No. of studies	Study design	Sample size	Risk of bias	Indirectness	Publication bias	Inconsistency	Imprecision	Effect estimate (95 % CI)	Heterogeneity (I^2^)	Certainty of evidence
6MWD	9	RCT	422	Serious①	Not serious	Likely	Not serious②	Not serious	MD 38.62 (25.72, 51.51)	0%	Moderate
FVC	6	RCT	321	Serious①	Not serious	Likely	Serious②	Not serious	SMD 0.35 (−0.11, 0.81)	75%	Low
FEV1	3	RCT	138	Serious①	Not serious	Likely	Serious②	Not serious	SMD 0.51 (−0.24, 1.25)	75%	Low
DLCO	3	RCT	176	Serious①	Not serious	Likely	Not serious②	Not serious	SMD 0.66 (0.35, 0.97)	26%	Moderate
SGRQ-Activity	4	RCT	151	Serious①	Not serious	Likely	Not serious②	Not serious	MD −12.01 (−18.63, −4.40)	0%	Moderate
SGRQ-Impact	4	RCT	151	Serious①	Not serious	Likely	Not serious②	Not serious	MD −11.11 (−17.60, −4.62)	41%	Low
SGRQ-Symptom	5	RCT	173	Serious①	Not serious	Likely	Not serious②	Not serious	MD −8.24 (−13.33, −3.15)	0%	Moderate
SGRQ-Total	6	RCT	261	Serious①	Not serious	Likely	Not serious②	Not serious	MD −7.75 (−12.13, −3.37)	44%	Low
Dyspnea Symptoms	5	RCT	204	Serious①	Not serious	Likely	Serious②	Not serious	SMD 0.12 (−0.76, 0.99)	88%	Low
Psychological Emotion	2	RCT	89	Serious①	Not serious	Likely	Serious②	Not serious	SMD −0.05 (−0.83, 0.73)	67%	Low
K-BILD-Total	2	RCT	89	Serious①	Not serious	Likely	Not serious②	Not serious	MD 1.37 (−3.51, 6.24)	0%	Moderate

①The random-sequence generation and allocation concealment were inadequate; there were deviations from the intended interventions or an open-label design; although missing data were handled with intention-to-treat analysis, outcome assessors were not blinded and selective outcome reporting was suspected. When ≥ 70 % of the participants contributing to an outcome come from studies judged to be at high risk of bias, GRADE downgrades the certainty of evidence by one level and records the domain as “serious risk of bias.” ②The pooled analysis showed statistically significant heterogeneity, which may compromise the robustness of the results.

## Discussion

This study synthesized data from 11 randomized controlled trials involving 503 IPF participants across 7 countries. Through systematic review and meta-analysis, it comparatively evaluated the efficacy of two primary structured exercise interventions—mind-body integration training and structured multi-component training—in patients with idiopathic pulmonary fibrosis. Results indicate that exercise interventions, particularly mind-body integrated training, may be associated with clear and relatively comprehensive benefits in improving patients’ exercise capacity, selected key pulmonary function measures, quality of life, and dyspnea symptoms. Furthermore, we rigorously assessed evidence quality using the GRADE framework to enhance the reliability of these findings for clinical decision-making.

Through meta-analysis, mind-body integrated training was associated with more favorable outcomes across several measures compared with structured multi-component training, which is consistent with the increasingly recognized concept of “integrated rehabilitation.” Regarding 6MWD, the improvement associated with mind-body integrated training (MD = 50.24 meters) was numerically larger than that of structured training (MD = 24.43 meters), although the subgroup difference reached only marginal statistical significance (*P* = 0.05). This finding lends partial support to the assertion by Spruit et al. (28) that “pulmonary rehabilitation should extend beyond isolated aerobic and resistance training to incorporate behavioral and psychological components for optimized overall function”. Furthermore, this meta-analysis showed that mind-body integrated training significantly improved both the total score and all domains of the SGRQ, suggesting a potentially broader impact on patients’ quality of life. These benefits may stem from the synergistic regulatory effects of such training on breathing patterns, body awareness, and psychological state (29).

Regarding pulmonary function, the significant improvement in FVC and DLCO achieved through mind-body integration training is particularly noteworthy. As a key predictor of disease progression in IPF, the stabilization or improvement of FVC holds significant clinical importance (30). The findings of this study align with a review of pulmonary rehabilitation by Dowman et al. (31), which indicated that training integrating respiratory control and relaxation techniques may be more effective in reducing dynamic overinflation, thereby creating space for improvement in lung volume. The improvement in DLCO suggests that such training may positively influence gas exchange efficiency across the alveolar-capillary membrane. Potential mechanisms include enhanced pulmonary blood flow distribution, reduced local inflammation, or optimized respiratory mechanics; however, further research is needed to confirm these effects (32).

The difference in efficacy between the two training methods may stem from their distinct mechanisms of action. Structured multi-component training (e.g., aerobic combined with resistance training) primarily targets the peripheral muscular system, enhancing exercise capacity by improving muscle strength, endurance, and metabolic efficiency. However, it may lack sufficient specificity for addressing core pathophysiological limitations in IPF patients, such as pulmonary mechanical constraints, impaired gas exchange, and fear-avoidance behaviors associated with dyspnea (33). In contrast, mind-body integration training (such as yoga, Baduanjin, and breath guidance) typically combines abdominal breathing, pursed-lip breathing, mental guidance, and slow, continuous movements. Its potential mechanisms of action may be multifaceted: (1) Optimized respiratory mechanics: Training reduces respiratory rate, increases tidal volume, decreases work of breathing, and reduces dynamic hyperinflation, thereby directly alleviating dyspnea and potentially indirectly improving lung volume parameters (34). (2) Autonomic Regulation: Mind-body training emphasizing deep, slow breathing and relaxation has been shown to enhance parasympathetic tone and reduce stress responses. This may help alleviate anxiety—a common condition in IPF patients—and the associated perception of breathlessness (35). (3) Neuromuscular Coordination and Postural Improvement: Training that focuses awareness on body posture and movement coordination may enhance the efficiency of accessory respiratory muscles and improve thoracic mobility, which is particularly important for patients with restrictive lung disease (36).

This study provides important implications for clinical practice. First, exercise intervention should be regarded as a safe and effective adjunct to the comprehensive management of IPF (including anti-fibrotic drug therapy). Second, when designing exercise prescriptions, priority should be given to incorporating mind-body integration training components. This approach aims to enhance exercise endurance while comprehensively improving patients’ respiratory sensations, quality of life, and select pulmonary function measures. For moderate-to-severe patients unable to participate in high-intensity training, low-intensity mind-body exercises emphasizing respiratory control and relaxation may represent a more feasible and better-tolerated entry point (37).

Although this study yielded certain findings, its limitations must be acknowledged: (1) First, limitations in evidence quality: The GRADE assessment indicated that the majority of outcome evidence was of “low” or “moderate” quality. This was primarily due to the widespread risk of bias in the included studies (e.g., inadequate randomization and blinding) and significant heterogeneity in some measures (e.g., FVC, dyspnea scores). This constrained our ability to draw definitive conclusions. (2) Heterogeneity in intervention protocols: Even within the same subgroup, specific training regimens varied, potentially confounding efficacy comparisons. Given insufficient sample sizes, we minimized this impact by comparing different training durations. (3) Sample size and population representativeness: The overall sample size remains relatively limited, and participants predominantly comprised elderly patients with mild-to-moderate IPF. Extrapolating results to broader populations (e.g., severe patients, different ethnicities) requires caution. (4) Lack of long-term follow-up data: Most studies had intervention periods of 8–12 weeks, lacking data on long-term efficacy and adherence. This prevents assessment of exercise’s sustained benefits and potential impact on disease progression. (5) A key limitation of this review is the indirect, observational nature of the subgroup analyses comparing mind-body integration with structured multi-component training. The lack of randomized, head-to-head comparisons means that any observed differences in effect estimates could be due to confounding factors (e.g., baseline disease severity, intervention duration, cultural context) rather than the training modality itself. Furthermore, most subgroup differences were not statistically significant, and those with *P*-values near 0.05 were often accompanied by high subgroup heterogeneity (I^2^ > 70%). Therefore, we strongly caution against interpreting these findings as evidence of superiority of one approach over the other. They serve to inform the design of future comparative efficacy trials, not to guide definitive clinical choice. (6) Additionally, adverse events were reported in detail in only one of the included studies. Therefore, the conclusion that all forms of structured exercise interventions have a high safety profile is not sufficiently supported by the current evidence. The absence of adverse event reporting in most trials may reflect either a true lack of events or, more likely, incomplete monitoring or reporting. (7) Furthermore, substantial heterogeneity was observed in the meta-analysis of dyspnea symptoms (I^2^ = 88%). This is likely attributable, at least in part, to the pooling of different dyspnea measurement instruments (L-IPF, mMRC, Dyspnea, BDI), which capture distinct constructs of breathlessness. Although standardized mean differences were used to accommodate unit differences, conceptual heterogeneity across scales remains a concern and may limit the interpretability of the pooled estimate.

To address the current evidence gap, future research should focus on conducting high-quality, large-sample randomized controlled trials that strictly adhere to the CONSORT reporting guidelines. These trials should implement thorough randomization, allocation concealment, and outcome assessor blinding to minimize bias risks (38). Furthermore, studies should standardize and comprehensively report exercise protocols using checklists such as TiDRaF or CERT to facilitate replication, comparison, and meta-analysis of results (39). Furthermore, it is essential to conduct in-depth investigations into the underlying mechanisms and individualized responses. By integrating respiratory mechanics, cardiopulmonary exercise testing, biomarkers, and neuroimaging techniques, we should elucidate the physiological and psychological pathways associated with different exercise modalities. This research aims to identify biomarkers capable of predicting treatment efficacy, thereby advancing the development of personalized exercise prescriptions. Additionally, long-term efficacy and cost-effectiveness must be evaluated. Long-term follow-up studies lasting ≥ 1 year should be designed to clarify the long-term impact of exercise intervention on acute exacerbations, hospitalization rates, and survival rates. Health economic evaluations should also be conducted to provide decision-making support for the promotion of rehabilitation strategies. Finally, it is necessary to develop and validate assessment tools specific to IPF, moving beyond existing generic or COPD-oriented scales. Establishing patient-reported outcome measures (PROMs) tailored to the unique characteristics of IPF will enable precise measurement of intervention effectiveness (40).

## Conclusion

Existing evidence suggests, that structured exercise interventions, particularly mind-body integration training that combines breathing, movement, and mental focus, may be potentially effective strategies for improving exercise capacity, quality of life, and certain pulmonary functions in IPF patients. However, given that most included studies were at high risk of bias and the overall evidence quality was low to moderate, these findings provide only preliminary, hypothesis-generating support for incorporating mind-body integration training into comprehensive rehabilitation management for IPF. Future research urgently requires more rigorous study designs, including high-quality randomized controlled trials with blinded outcome assessment, to verify these potential benefits, further clarify their efficacy, explore underlying mechanisms, and promote the implementation of personalized, highly accessible exercise rehabilitation programs in clinical practice.

## Data Availability

The original contributions presented in this study are included in this article/Supplementary material, further inquiries can be directed to the corresponding authors.
