# Supplementary material for: Comparative efficacy of different types of structured exercise interventions for idiopathic pulmonary fibrosis: a systematic review and meta-analysis
Source: Front Med (Lausanne). 2026 Jun 12;13:1875852. doi: 10.3389/fmed.2026.1875852 (PMC13303502; doi:10.3389/fmed.2026.1875852)
Supplement: Supplementary file 1 [file Table_1.docx]

**Search Strategy**

**Database——Pubmed**

| Search | Query | Results |
| --- | --- | --- |
| #1 | Tai Ji[MeSH Terms] or Exercise[MeSH Terms] or Exercise Therapy[MeSH Terms] or Sports or Motor Activity[MeSH Terms] or Exercise Movement Techniques[MeSH Terms] or Resistance Training[MeSH Terms] or Endurance Training[MeSH Terms] or Walking[MeSH Terms] or Running[MeSH Terms] or Bicycling[MeSH Terms] or Swimming[MeSH Terms] or Jogging[MeSH Terms] or Yoga[MeSH Terms] | 799,520 |
| #2 | Health Qigong[Title/Abstract] OR Baduanjin[Title/Abstract] OR Wuqinxi[Title/Abstract] OR Tai Ji[Title/Abstract] OR Yijinjing[Title/Abstract] OR Liuzijue[Title/Abstract] OR Ditangquan[Title/Abstract] OR Tai Chi[Title/Abstract] OR Mind–Body Exercise*[Title/Abstract] OR Mind–Body Therap*[Title/Abstract] OR Exercise[Title/Abstract] OR Exercise Therap*[Title/Abstract] OR Sport*[Title/Abstract] OR Physical Activit*[Title/Abstract] OR Motor Activit*[Title/Abstract] OR Exercise Movement Technique*[Title/Abstract] OR Aerobic Exercise[Title/Abstract] OR Resistance Training[Title/Abstract] OR Endurance Training[Title/Abstract] OR Walking[Title/Abstract] OR Running[Title/Abstract] OR Bicycling[Title/Abstract] OR Swimming[Title/Abstract] OR Jogging[Title/Abstract] OR Yoga[Title/Abstract] OR Pilates[Title/Abstract] | 808,853 |
| #3 | (Health Qigong[Title/Abstract] OR Baduanjin[Title/Abstract] OR Wuqinxi[Title/Abstract] OR Tai Ji[Title/Abstract] OR Yijinjing[Title/Abstract] OR Liuzijue[Title/Abstract] OR Ditangquan[Title/Abstract] OR Tai Chi[Title/Abstract] OR Mind–Body Exercise*[Title/Abstract] OR Mind–Body Therap*[Title/Abstract] OR Exercise[Title/Abstract] OR Exercise Therap*[Title/Abstract] OR Sport*[Title/Abstract] OR Physical Activit*[Title/Abstract] OR Motor Activit*[Title/Abstract] OR Exercise Movement Technique*[Title/Abstract] OR Aerobic Exercise[Title/Abstract] OR Resistance Training[Title/Abstract] OR Endurance Training[Title/Abstract] OR Walking[Title/Abstract] OR Running[Title/Abstract] OR Bicycling[Title/Abstract] OR Swimming[Title/Abstract] OR Jogging[Title/Abstract] OR Yoga[Title/Abstract] OR Pilates[Title/Abstract]) OR (Tai Ji[MeSH Terms] or Exercise[MeSH Terms] or Exercise Therapy[MeSH Terms] or Sports or Motor Activity[MeSH Terms] or Exercise Movement Techniques[MeSH Terms] or Resistance Training[MeSH Terms] or Endurance Training[MeSH Terms] or Walking[MeSH Terms] or Running[MeSH Terms] or Bicycling[MeSH Terms] or Swimming[MeSH Terms] or Jogging[MeSH Terms] or Yoga[MeSH Terms]) | 1,162,832 |
| #4 | Pulmonary Fibrosis[MeSH Terms] or Idiopathic Pulmonary Fibrosis[MeSH Terms] or Lung Diseases, Interstitial[MeSH Terms] | 91,970 |
| #5 | Pulmonary Fibrosis[Title/Abstract] OR Pulmonary Interstitial Fibrosis[Title/Abstract] OR Idiopathic Pulmonary Fibrosis[Title/Abstract] OR Virus-Related Pulmonary Fibrosis[Title/Abstract] OR Viral Pulmonary Fibrosis[Title/Abstract] OR Usual Interstitial Pneumonia[Title/Abstract] OR Diffuse Interstitial Pulmonary Fibrosis[Title/Abstract] OR Diffuse Pulmonary Fibrosis[Title/Abstract] OR Lung Diseases, Interstitial[Title/Abstract] | 31,840 |
| #6 | (Pulmonary Fibrosis[MeSH Terms] or Idiopathic Pulmonary Fibrosis[MeSH Terms] or Lung Diseases, Interstitial[MeSH Terms]) OR (Pulmonary Fibrosis[Title/Abstract] OR Pulmonary Interstitial Fibrosis[Title/Abstract] OR Idiopathic Pulmonary Fibrosis[Title/Abstract] OR Virus-Related Pulmonary Fibrosis[Title/Abstract] OR Viral Pulmonary Fibrosis[Title/Abstract] OR Usual Interstitial Pneumonia[Title/Abstract] OR Diffuse Interstitial Pulmonary Fibrosis[Title/Abstract] OR Diffuse Pulmonary Fibrosis[Title/Abstract] OR Lung Diseases, Interstitial[Title/Abstract]) | 102,509 |
| #7 | Randomized Controlled Trial[Publication Type] | 654,046 |
| #8 | #3 AND #6 AND #7 | **128** |

**Database——Web of Science**

((TS=(Health Qigong or Baduanjin or wuqiangxi or Tai Ji or Yijinjing or Liuzijue or dayangquan or Tai Chi or Mind–Body Exercise* or Mind–Body Therap* or Exercise or Exercise Therap* or Sport* or Physical Activit* or Motor Activit* or Exercise Movement Technique* or Aerobic Exercise or Resistance Training or Endurance Training or Walking or Running or Bicycling or Swimming or Jogging or Yoga or Pilates)) AND TS=(Pulmonary Fibrosis or Pulmonary Interstitial Fibrosis or Idiopathic Pulmonary Fibrosis or Virus-Related Pulmonary Fibrosis or Viral Pulmonary Fibrosis or Usual Interstitial Pneumonia or Diffuse Interstitial Pulmonary Fibrosis or Diffuse Pulmonary Fibrosis or Lung Diseases, Interstitial)) AND DT=(Article) | **476** results

**Database——Scopus**

( ( TITLE-ABS-KEY ( Health Qigong ) OR TITLE-ABS-KEY ( Baduanjin ) OR TITLE-ABS-KEY ( Wuqinxi ) OR TITLE-ABS-KEY ( Tai Ji ) OR TITLE-ABS-KEY ( Yijinjing ) OR TITLE-ABS-KEY ( Liuzijue ) OR TITLE-ABS-KEY ( Ditangquan ) OR TITLE-ABS-KEY ( Tai Chi ) OR TITLE-ABS-KEY ( Mind–Body Exercise* ) OR TITLE-ABS-KEY ( Mind–Body Therap* ) OR TITLE-ABS-KEY ( Exercise ) OR TITLE-ABS-KEY ( Exercise Therap* ) OR TITLE-ABS-KEY ( Sport* ) OR TITLE-ABS-KEY ( Physical Activit* ) OR TITLE-ABS-KEY ( Motor Activit* ) OR TITLE-ABS-KEY ( Exercise Movement Technique* ) OR TITLE-ABS-KEY ( Aerobic Exercise ) OR TITLE-ABS-KEY ( Resistance Training ) OR TITLE-ABS-KEY ( Endurance Training ) OR TITLE-ABS-KEY ( Walking ) OR TITLE-ABS-KEY ( Running ) OR TITLE-ABS-KEY ( Bicycling ) OR TITLE-ABS-KEY ( Swimming ) OR TITLE-ABS-KEY ( Jogging ) OR TITLE-ABS-KEY ( Yoga ) OR TITLE-ABS-KEY ( Pilates ) ) ) AND ( ( TITLE-ABS-KEY ( Pulmonary Fibrosis ) OR TITLE-ABS-KEY ( Pulmonary Interstitial Fibrosis ) OR TITLE-ABS-KEY ( Idiopathic Pulmonary Fibrosis ) OR TITLE-ABS-KEY ( Virus-Related Pulmonary Fibrosis ) OR TITLE-ABS-KEY ( Viral Pulmonary Fibrosis ) OR TITLE-ABS-KEY ( Usual Interstitial Pneumonia ) OR TITLE-ABS-KEY ( Diffuse Interstitial Pulmonary Fibrosis ) OR TITLE-ABS-KEY ( Diffuse Pulmonary Fibrosis ) OR TITLE-ABS-KEY ( Lung Diseases , Interstitial ) ) ) AND ( ( TITLE-ABS-KEY ( randomized controlled trial ) OR TITLE-ABS-KEY ( random ) ) ) | **737** results

**Database——Cochrane library**

| Search | Query | Results |
| --- | --- | --- |
| #1 | MeSH descriptor: [Qigong] explode all trees | 191 |
| #2 | MeSH descriptor: [Tai Ji] explode all trees | 632 |
| #3 | MeSH descriptor: [Mind-Body Therapies] explode all trees | 10130 |
| #4 | MeSH descriptor: [Exercise] explode all trees | 41560 |
| #5 | MeSH descriptor: [Exercise Therapy] explode all trees | 23860 |
| #6 | MeSH descriptor: [Sports] explode all trees | 23344 |
| #7 | MeSH descriptor: [Motor Activity] explode all trees | 45301 |
| #8 | MeSH descriptor: [Exercise Movement Techniques] explode all trees | 3880 |
| #9 | MeSH descriptor: [Endurance Training] explode all trees | 236 |
| #10 | MeSH descriptor: [Walking] explode all trees | 8629 |
| #11 | MeSH descriptor: [Running] explode all trees | 3010 |
| #12 | MeSH descriptor: [Bicycling] explode all trees | 2227 |
| #13 | MeSH descriptor: [Swimming] explode all trees | 672 |
| #14 | MeSH descriptor: [Jogging] explode all trees | 72 |
| #15 | MeSH descriptor: [Yoga] explode all trees | 1301 |
| #16 | (Baduanjin or Wuqinxi or Yijinjing or Liuzijue or Ditangquan or Mind–Body Exercise* or Physical Activit* or Aerobic Exercise or Resistance Training or Pilates):ti,ab,kw | 104020 |
| #17 | #1 or #2 or #3 or #4 or #5 or #6 or #7 or #8 or #9 or #10 or #11 or #12 or #13 or #14 or #15 or #16 | 146035 |
| #18 | MeSH descriptor: [Pulmonary Fibrosis] explode all trees | 952 |
| #19 | MeSH descriptor: [Idiopathic Pulmonary Fibrosis] explode all trees | 623 |
| #20 | MeSH descriptor: [Lung Diseases, Interstitial] explode all trees | 1934 |
| #21 | (Pulmonary Interstitial Fibrosis or Virus-Related Pulmonary Fibrosis or Viral Pulmonary Fibrosis or Usual Interstitial Pneumonia or Diffuse Interstitial Pulmonary Fibrosis or Diffuse Pulmonary Fibrosis):ti,ab,kw | 1041 |
| #22 | #18 or #19 or #20 or #21 | 2579 |
| #23 | #17 AND #22 in Trials | **183** |

**Database——Embase**

| Search | Query | Results |
| --- | --- | --- |
| #1 | 'tai chi'/exp OR 'qigong'/exp OR 'baduanjin'/exp OR 'wuqinxi'/exp OR 'yijinjing'/exp OR 'liuzijue'/exp OR 'mind body exercise'/exp OR 'exercise'/exp OR 'sport'/exp OR 'motor activity'/exp OR 'aerobic exercise'/exp OR 'physical activity'/exp OR 'resistance training'/exp OR 'endurance training'/exp OR 'walking'/exp OR 'running'/exp OR 'cycling'/exp OR 'swimming'/exp OR 'jogging'/exp OR 'yoga'/exp OR 'pilates'/exp OR 'kinesiotherapy'/exp | 1,860,382 |
| #2 | ditangquan:ti,ab,kw OR 'mind–body therap*':ti,ab,kw OR 'exercise therap*':ti,ab,kw OR 'exercise movement technique*':ti,ab,kw | 11,891 |
| #3 | #1 OR #2 | 1,861,283 |
| #4 | 'lung fibrosis'/exp OR 'fibrosing alveolitis'/exp OR 'usual interstitial pneumonia'/exp OR 'interstitial lung disease'/exp | 225,139 |
| #5 | 'pulmonary fibrosis':ti,ab,kw OR 'pulmonary interstitial fibrosis':ti,ab,kw OR 'idiopathic pulmonary fibrosis':ti,ab,kw OR 'virus-related pulmonary fibrosis':ti,ab,kw OR 'viral pulmonary fibrosis':ti,ab,kw OR 'diffuse interstitial pulmonary fibrosis':ti,ab,kw OR 'diffuse pulmonary fibrosis':ti,ab,kw OR 'lung diseases, interstitial':ti,ab,kw | 50,413 |
| #6 | #4 OR #5 | 229,669 |
| #7 | #5 AND #8 | 5,741 |
| #8 | #7 AND 'randomized controlled trial'/de | **373** |
